# Elucidating the Uptake and Distribution of Nanoparticles in Solid Tumors via a Multilayered Cell Culture Model

**DOI:** 10.1007/s40820-014-0025-1

**Published:** 2014-12-23

**Authors:** Darren Yohan, Charmainne Cruje, Xiaofeng Lu, Devika Chithrani

**Affiliations:** 1 Department of Physics, Ryerson University, 350 Victoria Street, Toronto, ON Canada; 2 Keenan Research Centre, Li Ka Shing Knowledge Institute, St. Michael’s Hospital, Toronto, ON Canada

**Keywords:** Gold nanoparticles, Tumor, Multicellular cell layers, Tissue

## Abstract

Multicellular layers (MCLs) have previously been used to determine the pharmacokinetics of a variety of different cancer drugs including paclitaxel, doxorubicin, methotrexate, and 5-fluorouracil across a number of cell lines. It is not known how nanoparticles (NPs) navigate through the tumor microenvironment once they leave the tumor blood vessel. In this study, we used the MCL model to study the uptake and penetration dynamics of NPs. Gold nanoparticles (GNPs) were used as a model system to map the NP distribution within tissue-like structures. Our results show that NP uptake and transport are dependent on the tumor cell type. MDA-MB-231 tissue showed deeper penetration of GNPs as compared to MCF-7 one. Intracellular and extracellular distributions of NPs were mapped using CytoViva imaging. The ability of MCLs to mimic tumor tissue characteristics makes them a useful tool in assessing the efficacy of particle distribution in solid tumors.

**Electronic supplementary material:**

The online version of this article (doi:10.1007/s40820-014-0025-1) contains supplementary material, which is available to authorized users.

## Introduction

**Fig. 1:**
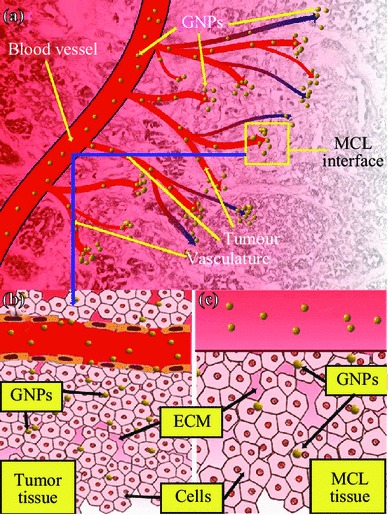
Use of MCL cell model to understand the NP transport through the tumor tissue. **a** Transport of GNPs through the blood vessels and enters tumor vasculature. The interface between tumor vasculature and tumor tissue is highlighted with a *yellow box*. **b** GNPs escape the tumor vasculature through leaky endothelial cells (1) and enter tumor cells through ECM. **c** Description shown in B is modeled using proposed MCL cell model. MCL act as a tumor tissue being fed by tissue culture media containing GNPs (2). (Color figure online)

Biomedical research on the use of materials called “nanoparticles” (NPs) for improved cancer imaging and therapy has come a long way [1–4]. Such nanotechnology platforms allow the development of cancer diagnosis and treatment that is more effective with reduced side effects [2, 3, 5–7]. The targeted delivery of treatments is the goal of NP systems so that safer methods are used [8–12]. Among NPs, research on gold nanoparticles (GNPs) plays a major role in cancer treatment because they can increase the damage inflicted by radiation and chemotherapeutic drugs, generate heat upon ultraviolet (UV) and near infrared refection (NIR) radiation exposure, and hence allow the eradication of cancer cells via thermal ablation, and therefore improve drug delivery for those that are water insoluble or unstable in vivo, and prolong the lifetime of drugs or imaging agents through NP surface modification, so that drug loss due to rapid clearance and metabolism is avoided [13–15]. For these reasons, GNPs are being studied as prospective therapeutic agents in cancer treatment options that include chemotherapy, radiation therapy (RT), photothermal therapy (PTT), and photodynamic therapy (PDT). These new findings encourage the development of effective combinational therapy in the battle against cancer [3]. The success of such innovations relies on GNP distribution and penetration throughout the tumor. To reach cancer cells in optimal quantities, therapeutic agents must be delivered to tumors through an imperfect blood vascular system cross vessel walls into the interstitium followed by penetrating multiple layers of tissue (see Fig. 1a, b) [16]. In this study, we used multicellular layers (MCLs) to simulate tumor tissue to study the penetration and uptake of GNPs in a tumor-like microenvironment.

The MCLs developed by Wilson and his colleagues provide a quantitative method that permits direct assessment of drug penetration through solid tissue [17, 18]. MCLs share several properties with solid tumors derived from the same cell type, including a similar but not identical extracellular matrix (ECM) and tight junctions between epithelial cells [19]. In addition, MCLs have been shown to exhibit areas of hypoxia, necrosis, as well as nutrient and proliferation gradients, and ECM generation [20–22]. The development of MCL models has facilitated quantification of drug penetration through solid tissue. Although the direct in vivo assessment, when feasible, has the advantage of duplicating the clinical environment most closely, in vitro techniques offer the advantage of being able to examine the distribution of agents of interest in the absence of complicating factors such as pharmacokinetics and hepatic metabolism which often differ between mice and humans [22].

**Fig. 2:**
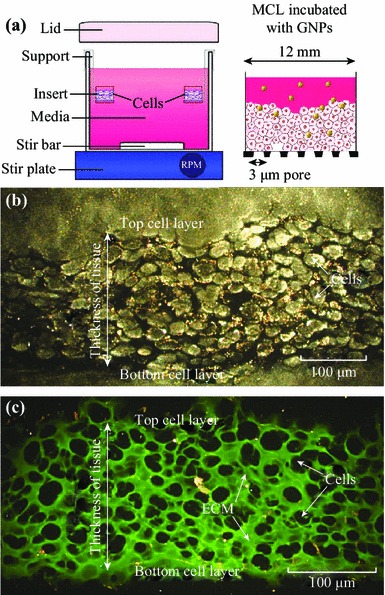
Growth of MCLs. **a** Diagrammatic representation of the apparatus used to culture MCLs. Tissue culture inserts are held suspended in stirred media (*top left*). The set-up was placed in a humidified incubator with 5 % O_2_, 5 % CO_2_, and 95 % N_2_. After the growth, GNPs were introduced into the media to investigate the NP transport through tissue (*top right*). **b** A cross-section of an unstained MCF-7 tissue. **c** A cross-section of a MCF-7 tissue stained with eosin to map the ECM. Areas marked in *green* belong to ECM, while the unstained regions represent cells. (Color figure online)

In this study, we examine for the first time the ability of NPs to penetrate and distribute through an MCL, which is designed to mimic the environment of solid tumor tissue (see Fig. 2). The MCL offers a model to study the transport of NPs across the tumor tissue once it leaves the blood vessel (Fig. 1c). The success of NP-based imaging and therapy depends on their delivery to tumor tissue through blood vessels as illustrated in Fig. 1a. It is known that NPs can leak out of tumor blood vessels and enter tumor tissue as illustrated in Fig. 1b. In this study, we grew tissue-like MCLs of up to 130–150 µm to study the NP transport and uptake in tumor tissue (Fig. 2b). Since the proliferation of tumor cells can outpace the proliferation of cells that form blood vessels, vascular density can be reduced, and the existence of cells as far as 100 µm from blood vessels is possible [23–25]. In addition, previous studies have shown that the presence of ECM in solid tumor tissue can affect the transport of molecules into tumor cells [26–28]. These MCLs would also consist of ECM which is similar but not identical to ones in solid tumors [19]. Hence, our MCL model creates a reasonable tumor microenvironment to study NP transport in tumor tissue. Elucidation of NP uptake and transport in tissue-like structures will bridge the gap between in vitro single-layer cell models and the in vivo tumor models.

## Materials and Methods

### Synthesis of GNPs

The GNPs were synthesized via the reduction of HAuCl_4_ by sodium citrate, which is more commonly referred to as the Turkevitch method [29]. By varying the amount of sodium citrate, the method can yield NPs of varying sizes. In this study, 20-nm particles were chosen since our future goal is to use these NPs for gene delivery. The GNPs were characterized by transmission electron microscopy (H7000; Hitachi Corp. Tokyo, Japan), UV-spectroscopy (Lambda 40; PerkinElmer, Waltham, MA), and by dynamic light scattering using 90 Plus Particle Sizer Analyzer (Brookhaven Instruments Corp. New York, NY) to determine the size of the particles.

### Growth of MCLs

The growth of the MCLs began with the growth of monolayer cells in a 5 % CO_2_ environment at 37 °C. After reaching confluence, these cells were trypsinized, centrifuged, suspended in media, and counted. Approximately, 150,000–200,000 cells were seeded onto a microporous membrane insert (Millicell, Bedford, MA). After allowing the cells to attach for 2–4 h, the inserts were washed with phosphate buffered saline (PBS) and then suspended in stirred media to grow. With pore sizes of 3 µm, the inserts allowed for the passage of stirred media through the base of the insert as seen in Fig. 2a.

Two breast cancer cell lines were used in this study: MCF-7 and MDA-MB-231. Cells were grown on the MCL insert in Dulbecco′s Modified Eagle′s Medium (LifeTechnologies Inc. Burlington, ON) with 10 % Fetal Bovine Serum (Sigma-Aldrich, Oakville, ON). Figure 2b is an image of an unstained tissue cross-section of MCF-7 cells. The ECM within the tissue was stained with eosin for visualization (Fig. 2c). The thickness of the tissue was controlled by the growth period. MCL incubation with NPs was done by hanging the MCLs in multiwall plates followed by filling the top of the inserts with the GNP and media mixture. A supply of fresh media was placed below the MCL to allow for GNPs that had penetrated the entire MCL structure to diffuse.

### Quantification of GNP Uptake

During incubation, the MCL structures were hung in multi-well plates with a 15 nM GNP/media mixture on the top and a supply of fresh media on the bottom. Both the ‘top’ and ‘bottom’ volumes were collected and measured for gold content via inductively coupled plasma-atomic emission spectroscopy (ICP-AES) (Optima 7300 DV; PerkinElmer, Waltham, MA). By using the known total concentration of GNPs, uptake and penetration through tissue may be measured, which results from taking the difference between the ‘top’ and ‘bottom’ samples. Monolayer cultures were also grown and harvested at three different time points or cell densities. These cultures were incubated with GNPs for 24 h and were used for cell counting and monolayer gold uptake measurement via ICP-AES. To determine the uptake as a function of layers, the difference in uptake between consecutive days of growth was observed (Fig. 8b). Because each new day of growth introduced new layers, the difference in uptake between consecutive days allowed for the quantification of uptake as a function of layer.

### Qualitative Analysis

To qualitatively measure the distribution of the GNPs as well as to provide a measure of MCL growth characteristics, MCL inserts were frozen in OCT compound for sectioning. The frozen MCLs were then sectioned (Cryostat CM1900; Leica, Wetzlar, Germany) into 15–20-µm-thick sections and placed onto slides for imaging. Tissue sections were stained with eosin to show the presence of ECM (Autostainer XL; Leica, Wetzlar, Germany). Stained tissue sections were imaged using the CytoViva Hyperspectral Imaging (HSI) dark-field microscope. By examining the images acquired by HSI (Fig. 8c, d), a qualitative examination of the layer-by-layer penetration was deduced.

### CytoViva Imaging of NP Distribution in MCLs

**Fig. 3:**
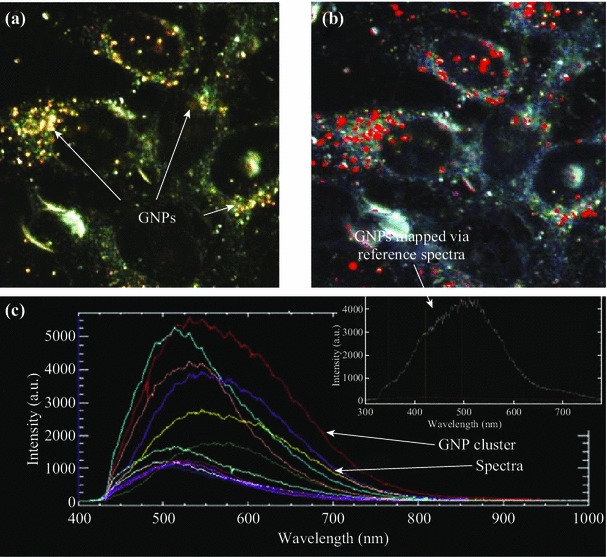
Visualization and mapping of GNPs in cells using CytoViva HSI optical microscopy. **a** The unmapped dark-field HSI image with GNPs visible as bright spots. **b** The result of a spectral angle mapping on the HSI image. GNPs have been labeled *red* as a result of matching spectra from individual pixels. **c** GNP spectra from few NP clusters localized within cells and the reference spectra (*inset*) used to create the spectral angle map in **b**. (Color figure online)

The CytoViva technology used in this study was specifically designed for optical observation and spectral confirmation of NPs as they interact with cells and tissues. The illumination of the microscope system utilizes oblique angle illumination to create high SNR dark-field images. Figure 3a is a dark-field image of a group of cells with internalized GNPs. The GNPs appear bright owing to their high scattering cross-section. HSI was used in conjunction with the dark-field microscope to obtain reflectance spectra from each pixel in the dark-field image. Spectral Angle Mapping can be performed to conduct a pixel-by-pixel matching of any spectra obtained by the system. This procedure was used to create a map of GNPs based on their reflectance spectra within the sample. The hyperspectral image shows which HSI pixels matched the known GNP spectrum within a given spectral angle threshold set at 0.15 radians for this study. Figure 3b shows the hyperspectral image with an overlaid spectral angle map where the red dots represent matching GNP spectra. Figure 3c shows reflectance spectra from one of the red dots and the reference spectrum (white color) to which pixels were matched. Reference spectrum was chosen from a sample of GNP spectra collected via the HSI image. It is representative of a typical GNP spectrum for the sample studied. The background reflectance spectra from the cytoplasm and ECM are shown in red. It can be clearly seen that the GNP clusters have a very distinct reflectance spectra compared to the background.

## Results and Discussion

In this study, we demonstrate for the first time the differences in GNP uptake between monolayer and tissue-like MCL models. GNPs were used as a radiation dose enhancer, anticancer drug carrier, and an imaging contrast agent in cancer research as discussed in the introduction. However, the success of NP-based imaging and therapy depends on the efficiency of delivery of NPs tumor tissue as illustrated in Fig. 1a. In this study, we investigated the NP transport across tumor tissues by using MCL cell model for the first time. It was successfully used to understand NP diffusion in tissues. The results were consistent with drug diffusion patterns observed in solid tumor in animal models.

It is known that NPs can leak out through the disorganized endothelial cells in tumor blood vessels and enter the tumor tissue (Fig. 1b**)**. In particular, this MCL model was used to study the transport of NPs across the tumor tissue once they leave blood vessels (Fig. 1c). The device used for growing MCLs is shown in Fig. 2a. A tissue cross-section of an approximately 150-µm-thick MCF-7 cell is shown in Fig. 2b and 2c. An unstained tissue cross-section is shown in Fig. 2b, while a tissue cross-section stained with eosin to highlight the ECM is shown in Fig. 2c. We used the CytoViva HSI technique to image the tissue and NPs. Unlike other optical imaging techniques, HSI allows us to map the GNPs via reflectance spectroscopy. This imaging technology does not require optically labeling NPs for their visualization. This is the first time that such imaging technology was used to visualize GNP distribution in tissue-like structures. Figure 3a shows the unmapped dark-field HSI image with GNPs visible as bright yellow spots, while Fig. 3b shows the result of spectral angle mapping on the HSI image. GNPs have been labeled red as a result of matching spectra (shown as an inset figure in Fig. 3c) from individual pixels. Figure 3c shows a few spectra from GNP clusters displayed in Fig. 3a. This imaging technique was used to map NP distribution through the tissue. Our first goal was to investigate the difference between monolayer and MCL cell models in terms of growth before investigating the NP uptake and transport.

**Fig. 4:**
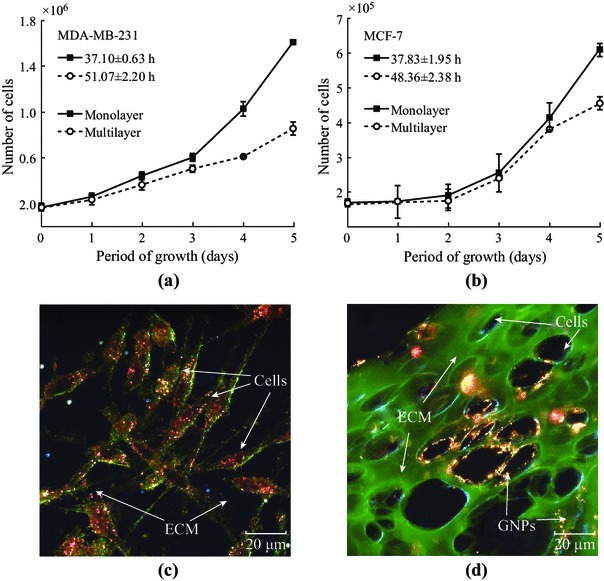
Characterization of monolayer and multilayer cell structures. **a**–**b** Comparison of growth curves for the MDA-MB-231 and MCF-7 cell lines at monolayer and multilayer level, respectively. **c**–**d** A monolayer and multilayer cross-section of MDA-MB-231 cells stained with eosin to highlight the ECM, respectively. Cell population doubling times for MCF-7 and MDA-MB-231 monolayer cell cultures were 38.83 and 37.10 h, respectively. Cell population doubling times for MCF-7 and MDA-MB-231 multilayer cell cultures were 48.36 and 51.07 h, respectively. *Error bars* represent the standard deviation and *n* = 3. There was no statistically significant difference via one-way ANOVA test between the monolayer and multilayer groups (*p* = 0.6907 for MCF-7 and *p* = 0.3751 for MDA-MB-231)

We monitored the growth of monolayer and multilayer cell samples over a period of time to understand the difference between these two cell models. As shown in Fig. 4, MCLs differ from the monolayer cultures in terms of growth and ECM generation. Both cell lines show an increase in population doubling time in the MCLs (Fig. 4a, b). For example, the population doubling time increased from 37 (at monolayer level) to 51 h (at multilayer level) for MDA-MB-231, while it increased from 39 (at monolayer level) to 48 h (at multilayer level) for MCF-7. Increase in population doubling time has previously been demonstrated for cell lines growing as solid tumors in vivo [30–32]. Though calculated doubling times were higher for the multilayer sample, there was no statistically significant difference via one-way analysis of variance (ANOVA) test between the monolayer and multilayer groups (*p* = 0.6907 for MCF-7 and *p* = 0.3751 for MDA-MB-231). Earlier work has shown that MCLs develop oxygen, proliferation, and nutrient gradients that lead to deficiencies in their availability across the MCL sample [20, 21, 33]. In the case of monolayer cell cultures, the uniform availability of oxygen and nutrients is likely a major contributor to the lower doubling time [30, 34, 35]. Figure 4c, d shows that there is significant increase in the presence of ECM (areas marked in green) in MCLs in contrast to monolayer cell cultures. The data are shown for the MDA-MB-231 cell line. Similar results were obtained for the MCF-7 cell line as well. There were major increases in the presence of ECM in the MCLs for both cell lines. ECM in the MCLs also appears thicker and more structured as compared to the monolayer ECM. In addition, GNPs (yellowish brown small dot-like structures) were mostly localized within cells in monolayer cell cultures (Fig. 4c). However, GNPs were localized in both the ECM and cells in the MCL cell cultures (Fig. 4d). Hence, one of the goals of this study is to understand how ECM affects NP transport at the tissue level.

**Fig. 5:**
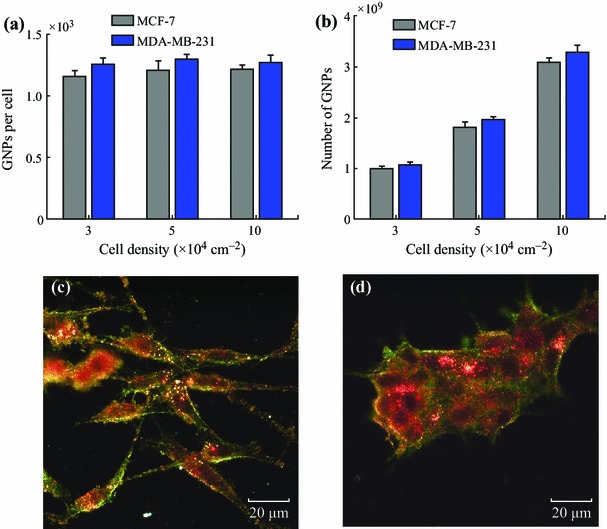
GNP uptake in monolayer cell models. **a** NP uptake per cell as a function of cell density. **b** Total uptake of NPs as a function of cell density. **c**–**d** Samples of H&E stained monolayer MDA-MB-231 and MCF-7 cells with GNPs present mostly in the cells. *Error bars* represent the standard deviation and *n* = 3. One-way ANOVA test over the cell densities for each cell line revealed that there was no significant difference in NP uptake for the MDA cell line (*p* = 0.565) and for the MCF-7 cell line (*p* = 0.3541)

We have also quantified NP uptake and transport in monolayer and MCL models. At the monolayer level, NP uptake per cell is mostly independent of cell density (Fig. 5). We performed a one-way ANOVA over cell densities for each cell line. This test revealed that there was no significant difference in the means for the MDA-MB231 cell line (*p* = 0.565) and for the MCF-7 cell line (*p* = 0.3541). A two-sample *t* test was also performed between the two cell lines at each density. These tests indicate that there is no significant difference in NP uptake between cell densities over the 3 trials performed at a 5 % significance level. This is likely due to the fact that GNPs introduced into monolayer cultures have immediate access to all cells thus enabling the efficient uptake of GNPs per cell. For example, NPs did not need to be transported through a dense ECM. Optical images showed that most of the NPs were localized within the cells, and the presence of ECM was minimal (Fig. 5b, c). In the next section, we will discuss the differences in ECM generated for MCF-7 and MDA-MB-231 cell lines before discussing NP transport through tissue structures.

**Fig. 6:**
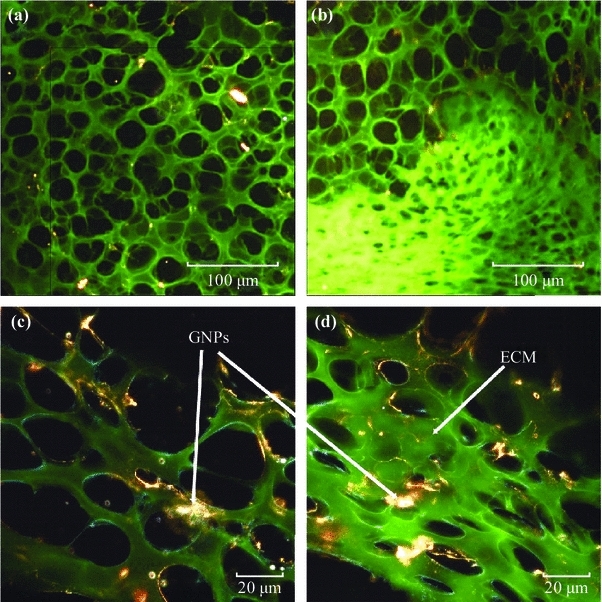
Differences in extracellular matrix (ECM) in MDA-MB-231 and MCF-7 tissue structures. **a**–**c**, MCL tissue of MCF-7 cells at ×10 and ×60 magnification, respectively. **b**–**d**, MCL tissue of MDA-MB-231 cells at ×10 and ×60, respectively. Differences in the ECM structure can be seen at both magnifications. MCF-7 tissue had a much more organized ECM structure, while MDA-MB-231 tissue has a disorganized ECM structure which allowed easy penetration of molecules into deeper tissues

The difference in ECM between the two MCL models (MCF-7 and MDA-MB-231) was made apparent in Fig. 6 where both were stained with eosin and appear green when viewed with a dark-field condenser. The ECM of MDA-MB-231 appears far less organized than the ECM of MCF-7. The ECM of MCF-7 presents ordered scaffolding with cells separated in distinct layers throughout the structure (Fig. 6a, c), whereas the cell layers are much less compartmentalized in the MDA-MB-231 ECM (Fig. 6b, d). It has been suggested that aggressively invasive tumor cells like MDA-MB-231 secrete matrix-degrading proteinases that serve to break down collagen [36–38]. The degradation of the collagen network in the ECM has been shown to significantly diminish the ability of the ECM to control the flow of interstitial traffic [26, 39–41]. This breakdown of ECM is not as apparent in the non-invasive MCF-7 cell line, and ECM will act as a barrier for NP transport. It is clear that ECM of different tumor cell lines can vary and these models will play a bigger role in optimizing NP-based therapeutic and imaging systems before moving into in vivo studies. In the next section, we discuss how the differences in ECM can affect NP transport through these tissue-like MCL models.

**Fig. 7:**
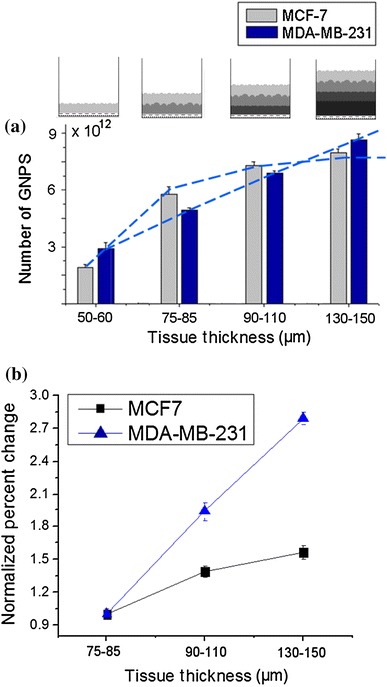
GNP uptake in multilayer cell models. **a** Accumulation of GNPs in tissue as a function of its thickness. NPs were able to penetrate deep into tissue in MDA-MB-231 tissue due to the breakdown in ECM matrix. In MCF-7 tissue, most NPs were localized at the top layers and properly organized ECM acted as a barrier for their transport deep into the tissue. **b** The normalized percent increase in GNP uptake as a function of tissue thickness. *Error bars* represent the standard deviation and *n* = 3

Our investigation of NP transport in tissue-like MCL models shows that NP penetration in tissue is dependent on the tumor cell line, possibly due to the differences in their ECM structure as discussed in the previous section (Fig. 7). At monolayer cell cultures, there was a linear increase in NP uptake as a function of cell density (Fig. 5). However, we noticed a deviation from this linear increase in NP uptake especially in MCF-7 multilayer structures (Fig. 7). Two-sample *t* test was used to compare the GNP uptake for each cell line at each thickness. The GNP uptake was found to be significantly different only for the smallest thickness (50–60 μm) with a *p* value of 0.047. For the other three thicknesses, the difference between the total NP uptake was not significant with *p* = 0.052 for 75–85 μm thickness, 0.063 for 90–110 μm thickness, and *p* = 0.091 for thickness greater than 130 μm. We attribute such difference to the fact that the lower thickness still allows for the clearance of GNPs in both models. Only when tissue is thicker does the effect become more pronounced due to its higher resistance to penetration. This reduction in uptake as a function of tissue depth is also apparent for a number of anticancer drugs, and is likely a major contributor to resistance to drugs in tumors [22, 42–44]. According to Fig. 7, the MCF-7 multilayer structures had a much higher resistance to GNP penetration as compared to the MDA-MB-231 MCL structure. This variation in NP penetration through MCLs generated from different cell lines could be mainly due to the differences in their ECM as discussed previously. In MDA-MB-231 tissue, NPs were able to penetrate deeper due to breakdown in the ECM matrix. However, MCF-7 tissue had a much more organized ECM, and therefore, ECM acted as a barrier to NP penetration.

**Fig. 8:**
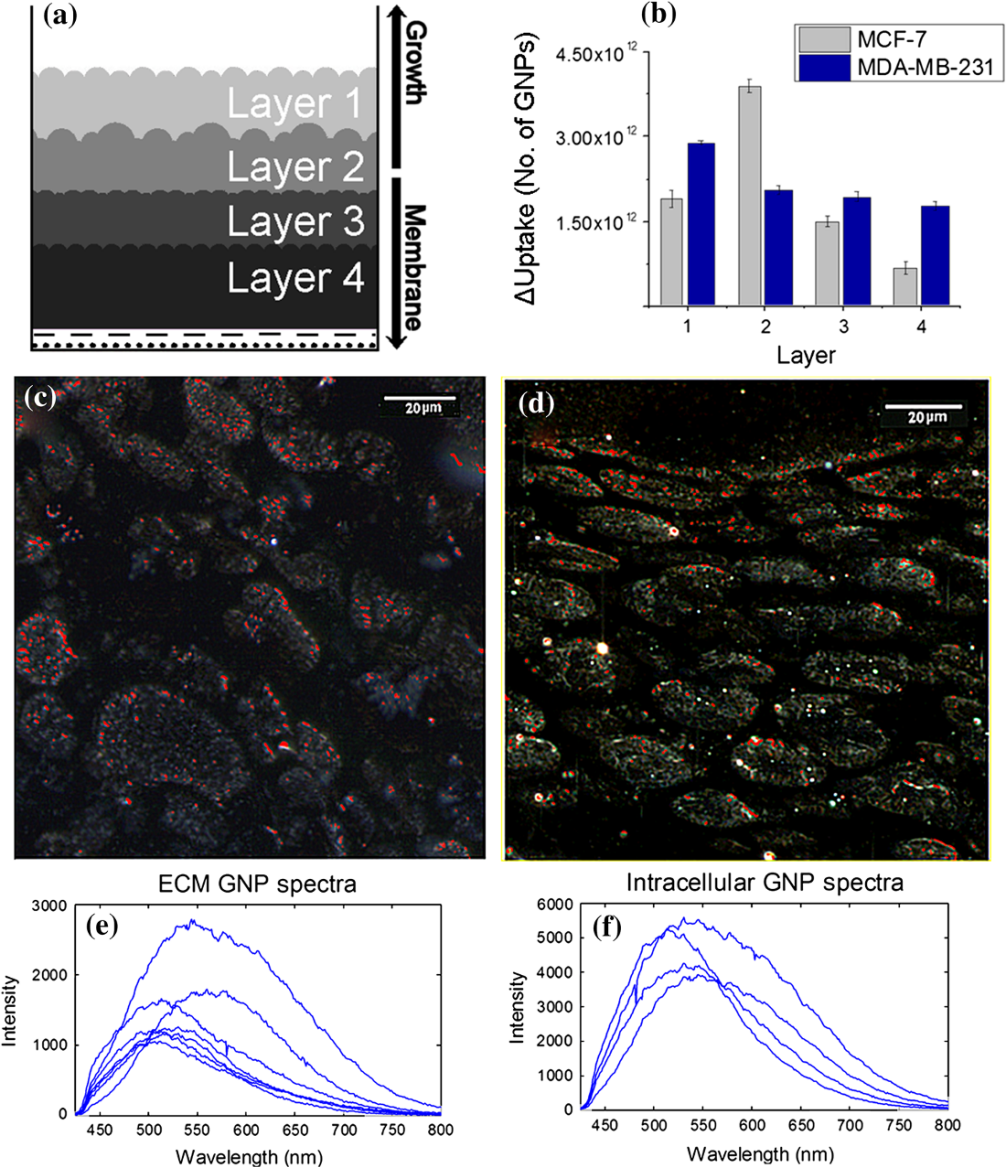
Mapping of NP transport through different layers of a thicker tissue. **a** A schematic depicting the multiple layers of a tissue cross-section. **b** Accumulation of NPs in different layers of tissue. **c**–**d** Mapping of the NP distribution in MDA-MB-231 and MCF-7 tissue (with GNPs labeled in *red*), respectively. **e**–**f** A sample of reflectance spectra of GNPs localized in ECM and cells, respectively. *Error bars* represent the standard deviation and *n* = 3. (Color figure online)

In the previous section, we discussed the accumulated transport of NPs in tissues with different thicknesses (Fig. 7). We were able to differentiate NP transport in different layers of a thicker tissue quantitatively and qualitatively (see Fig. 8). According to Fig. 8b, MDA-MB-231 tissue allows higher NP penetration in contrast to MCF-7. Figure 8c shows that MDA-MB-231 cells were able to access NPs despite greater tissue depth. Transport of NPs through MCF-7 tissue is illustrated in Fig. 8d and fewer were found a depth of the tissue. This further explains the outcome shown in Fig. 6. Using the CytoViva microscope, we looked at the spectral differences between NPs localized within the cell and in the ECM. The clustering of NPs in the cells is expected as GNPs are known to be grouped into endosomes upon entry into the cell via receptor-mediated endocytosis [45, 46]. According to Fig. 8e, f, NPs internalized by cells have an overall red shift in the spectra as compared to NPs in the ECM. This shows that most of the NPs in the ECM are still not aggregated as compared to ones within cells. Hence, it is important to optimize the transport of these NPs through ECM in order for them to reach cells effectively. This is the first time NP transport through tissue-like structures is studied using MCL cell models.

There is a tremendous effort to incorporate NPs into existing cancer therapeutic protocols. We have demonstrated that MCL model can be used to study the transport of novel NP-based systems to optimize their delivery to tumor tissue. Our future goal is to study how the size and shape of NPs affect their transport through the ECM using this MCL model. Our preliminary studies showed that smaller NPs display higher tissue penetration as compared to larger NPs (supplementary section S2). This result is consistent with previous in vivo data. For example, Puvanakrishnan et al. investigated the in vivo tumor targeting efficiency of pegylated gold nanoshells (GNSs) and gold nanorods (GNRs) for single and multiple dosing [47]. The results showed that the smaller GNRs accumulated in higher concentrations in the tumor in comparison to larger GNSs. Moreover, Zang et al. have shown that 20-nm GNPs showed significantly higher tumor uptake than 40- and 80-nm GNPs [48]. These in vivo studies clearly show that smaller NPs transport easily through the ECM in comparison to larger NPs. The importance of this study is that we showed that MCL model could be used to mimic NP transport in tumor-like environments. Our results are consistent with published in vivo data. This model can be used to understand the transport and therapeutic response of NP complexes prior to use in animal models. Our future goal is to study how NP size and shape affect their transport in vivo and correlate their transport within the tumor tissue using our in vitro MCL model.

## Conclusions

This work demonstrates the importance of understanding the limitations of monolayer cultures in their ability to predict the uptake and effectiveness of cancer treatments in solid tumors. Furthermore, these results underscore the importance of the ECM in terms of throughout tumor tissue. By engaging these issues, GNP-based systems can be designed for combined chemotherapy and radiation therapy to better overcome the resistance to drugs and radiation found in solid tumors [3]. This would accelerate such NP-based innovations into clinics for the improved quality of life of cancer patients.

## Electronic supplementary material

Below is the link to the electronic supplementary material.

Supplementary material 1 (PDF 170 kb)
