# Supplementary material for: Elucidating the Uptake and Distribution of Nanoparticles in Solid Tumors via a Multilayered Cell Culture Model
Source: Nanomicro Lett. 2014 Dec 23;7:127–37. doi: 10.1007/s40820-014-0025-1 (PMC6223939; doi:10.1007/s40820-014-0025-1)
Supplement: Supplementary file 1 — Supplementary material 1 (PDF 170 kb) [file 40820_2014_25_MOESM1_ESM.pdf]

Supporting Information for

Elucidating the Uptake and Distribution of Nanoparticles in Solid Tumors *via* a Multilayered Cell Culture Model

Darren Yohan[1], Charmainne Cruje[1], Xiaofeng Lu[2], Devika Chithrani[1,2,]*

[1]Department of Physics, Ryerson University, 350 Victoria Street, Toronto, ON, Canada, M5B 2K3

[2]Keenan Research Centre, Li Ka Shing Knowledge Institute, St. Michael's Hospital, Toronto, ON, Canada.

*Corresponding author. E-mail address: devika.chithrani@ryerson.ca

Supplementary Section S1

Tranmission Electron Microscopy Image of Gold Nanoparticles Used for the Study

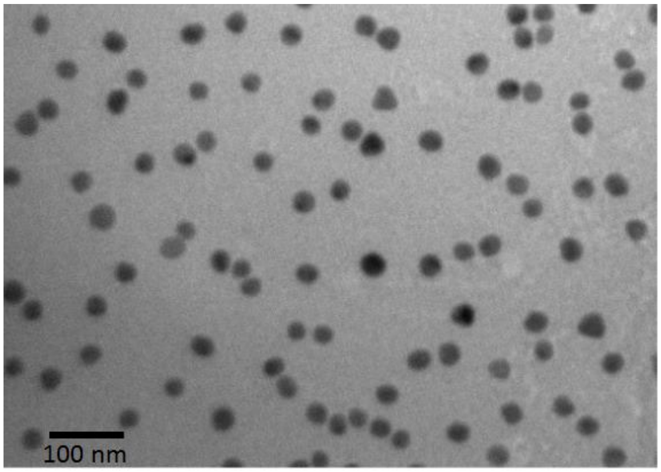

Fig. S1 Transmission Electron Microcopy image of GNPs

A smaller droplet of GNPs was spread onto carbon-coated copper grids. Dried copper grids were imaged using Hitachi H7000 transmission electron microscope (TEM; Hitachi Corp., Tokyo, Japan). To analyze the size distribution, the TEM micrographs were segmented in MATLAB. Five batches of 20 NPs were selected for analysis of NP size. Particle size was reported as the approximate diameter of NPs. The mean diameter was 19.80 ± 3.20 nm.

Supplementary Section S2

Effect of NP Size on Their Distribution Through the Tissue-like MCL Structures

Our preliminary data showed that 20 nm GNPs had a higher tissue penetration as compared to 50 nm GNPs

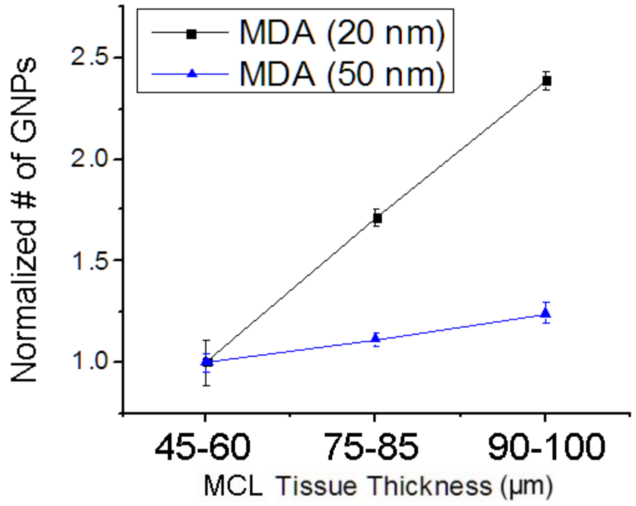

Fig. S2 Preliminary results comparing the uptake of 50 nm particles with 20 nm particles in MDA-MB-231 cells. The 50 nm particles show a much lower uptake as a function of tissue thickness than the 20 nm particles over the MDA-MB-231 cell line. Error bars represent the standard deviation over 3 ICP measurements.
